# Music and literature: are there shared empathy and predictive mechanisms underlying their affective impact?

**DOI:** 10.3389/fpsyg.2015.01250

**Published:** 2015-08-24

**Authors:** Diana Omigie

**Affiliations:** Music Department, Max Planck Institute for Empirical Aesthetics, Frankfurt am Main, Germany

**Keywords:** music, literature, emotions, esthetic, empathy, theory of mind, tension, active inference

## Abstract

It has been suggested that music and language had a shared evolutionary precursor before becoming mainly responsible for the communication of emotive and referential meaning respectively. However, emphasis on potential differences between music and language may discourage a consideration of the commonalities that music and literature share. Indeed, one possibility is that common mechanisms underlie their affective impact, and the current paper carefully reviews relevant neuroscientific findings to examine such a prospect. First and foremost, it will be demonstrated that considerable evidence of a common role of empathy and predictive processes now exists for the two domains. However, it will also be noted that an important open question remains: namely, whether the mechanisms underlying the subjective experience of uncertainty differ between the two domains with respect to recruitment of phylogenetically ancient emotion areas. It will be concluded that a comparative approach may not only help to reveal general mechanisms underlying our responses to music and literature, but may also help us better understand any idiosyncrasies in their capacity for affective impact.

## Introduction

The creation, performance, and consumption of music and literary works are preoccupations present in all cultures. Music and literature (in its original form of storytelling) have ancient origins and do not only lie at the heart of religious and cultural practices and narratives, but also provide widely popular leisure activities in everyday life. A number of psychological theories, some building on ideas dating as far back as classical antiquity (Aristotle, 1961; Longinus, 1965), have sought to account for music and literature’s affective capacity independently (Meyer, 1956; Goldman, 2006; Huron, 2006; Zunshine, 2006; Keen, 2007). Others have examined the nature of emotion in response to the arts more generally (Hjort and Lavers, 1997; Robinson, 2007). Most accounts, however, maintain that in spite of any differences in propositional content (Slevc and Patel, 2011), and notwithstanding any prevailing notions regarding evolutionary capacity for emotion (Brown, 2000), both music and literary works share a considerable ability to evoke powerful feelings. Further, evidence that music may evoke semantic representations (Koelsch et al., 2004; Steinbeis and Koelsch, 2008) would seem to temper any strong claims that the two are incomparable in terms of their ability to convey meaning.

Perhaps one of the most important qualities that binds music and literary reading, and differentiates them from a number of other cultural artifacts (such as paintings and sculpture), is that both unfold in time, offering a kind of “narrative” that can be followed (Rabkin, 1973; Maus, 1991; Levinson, 2004). While poetry, like music, may exercise affective impact through its emphasis on temporal stress and repetition, many different accounts emphasize the impact that even non-versed literary forms (e.g., short stories and novels) can have (Oatley, 1994; Hogan, 2010). Thus, given the timeless and universal appeal of music and literary storytelling, not to mention the claims from a range of theoretical accounts, it seems relevant to explore the emerging neuroscience research for any evidence of an overlap in underlying affective mechanisms. Recent research into music and literature has investigated a breadth of issues varying in their degree of domain specificity (e.g., Bohrn et al., 2012), and a comprehensive review of all possible links that can be made between the two art forms would require a longer format. Accordingly, the current perspective article focuses solely on what are considered key lines of investigation that have seen significant interest in both domains: namely, music and literature’s invocation of empathy and predictive processes and the potential role these mechanisms may play in emotion induction.

## Inferring and Sharing Emotions

The notion of empathy-like mechanisms being involved in literary response is one that dates as far back as Aristotle’s Poetics (Aristotle, 1961). Similarly, as early as in the eighteenth century, it was suggested that engagement of children with music is especially valuable in teaching emotions and a good social attitude. Today, in neuroscience and psychology, empathy may be broadly defined as the ability to infer and share emotional experiences (Gallese, 2003). It is held to comprise two different components: a cognitive and an emotional one that encompass the notion of perspective-taking and shared visceral feeling respectively (Shamay-Tsoory et al., 2009). Importantly, while the former is related to the notion of Theory of Mind (TOM) and mentalizing (Frith, 1999), the latter is seen as coinciding with the notion of emotional contagion (Juslin and Västfjäll, 2008; Juslin, 2013), with evidence of a double dissociation observable in the neuroscience literature (Eslinger, 1998; Shamay-Tsoory et al., 2003; Schulte-Ruther et al., 2007).

Fiction, the form of literature seen in short stories and novels, has been described as a kind of simulation of the social world (Mar and Oatley, 2008) and it has been suggested that its invocation of social situations not only explains readers’ tendency to mentalize during reading (Gygax et al., 2003) but also to feel emotions themselves (Cupchik et al., 1998; Miall and Kuiken, 2002; Oatley, 2002). Over the years, a large body of neuroimaging studies has focused on the neural correlates of text comprehension (see Mar, 2011, for a review), emotion processing in single words (Citron, 2012) and perspective-taking (van Overwalle, 2009). However, the extent to which cognitive or emotional empathy could be directly linked to the affective impact of literature remained limited. In recent years, however, it is becoming apparent that as text stimuli are rendered increasingly story-like or as feelings and emotions play a larger role in them (in other words, as text stimuli begin to resemble fiction or narrative literature), increasingly recruited are not only those areas involved in TOM processing [e.g., ventromedial prefrontal cortex (vmPFC) and temporoparietal junction (TPJ)] but also limbic or emotion areas like the amygdala, thalamus, and orbitofrontal cortex (OFC; Wallentin et al., 2011). Indeed, in line with the notion of emotion induction occurring as a consequence of perspective-taking during literary reading, TOM areas and structures like the amygdala have been implicated in narrative contexts concerning characters’ feelings (Ferstl et al., 2005), in negatively valenced stories (Altmann et al., 2012), in emotional relative to non-emotional sections of *Harry Potter* (Hsu et al., 2014) and when participants heard spoken narratives describing real-life emotional episodes (Nummenmaa et al., 2014). A recent series of studies has provided further compelling evidence that the greater the emotional content of a story, the greater the recruitment of both cognitive and emotional empathy-related structures such as anterior insula and mid cingulate (Altmann et al., 2012; Hsu et al., 2014, 2015a,b,c). Such findings are in line with the so-called *fiction feeling hypothesis* (Jacobs, 2015), which states that greater emotionality in a narrative results in greater feelings of empathy and immersion.

In music, several studies have implicated various limbic and paralimbic structures in the processing of basic emotions, arousal and valence (e.g., happy vs neutral and consonance vs dissonance; see Koelsch, 2014, for a review). However, it may be argued that since music is not itself an emotional object, at least some emotions induced while listening to it must be inferred (Downey et al., 2013). Supporting the notion that musical emotion may be inferred is the evidence that listeners show activation in structures associated with cognitive empathy during music listening. Steinbeis and Koelsch (2009) showed that when music listeners believed they were listening to a piece of music composed by a human rather than a computer, brain areas typically involved in mentalising, such as the medial prefrontal cortex (mPFC), were activated. Further, in the condition known as Behavioral variant frontotemporal dementia, which is associated with a large network of structures including those involved in mentalising, it was shown that the mentalising deficits normally exhibited by these patients also extended to the music domain (Downey et al., 2013). Specifically, patients were impaired in attributing mental states (e.g., dreamy), but not non-mental characteristics (e.g., raindrops) to music, with performance on the former task being more strongly associated with the vmPFC. Recent evidence of the recruitment of the default mode network (DMN) while listeners listened to their preferred music (Kay et al., 2012; Wilkins et al., 2014) also begs the question of the extent to which mentalising processes determine music preferences. The DMN is a network of structures that is preferentially activated when individuals engage in internal tasks like mind wandering and imagining the future. Critically, however, its sharing of a key structure, the mPFC, with the empathizing network, has been used to explain its frequent recruitment during mentalising and empathizing tasks (Gusnard et al., 2001; Li et al., 2014).

In general, while it may seem highly plausible that readers empathize with human characters in a literary work, the notion of music-evoked empathy has tended to be less intuitive. It is therefore worth noting that in addition to the evidence obtained using neuroscience techniques, numerous behavioral and physiological studies continue to provide persuasive support for the role of empathy-related processes during music listening. For instance, it has been reported that the strength of emotions induced in music listeners (self-report and physiology) modulates as a function of perspective-taking with the music performer (Miu and Baltes, 2012). Further, it has also been suggested that the discrepancy that sometimes exists between *expressed* and *felt* emotions (Gabrielsson, 2002) may be explained by the subjective degree of empathy felt by the listener for the musician (Egermann and Adams, 2013). Finally, it has been argued that the degree of the empathy trait possessed by a music listener may predict their appreciation of sad music (Taruffi and Koelsch, 2014).

Thus, taken together, a growing body of behavioral, physiological and neuroscience research provides support for the longstanding notion that empathy processes may contribute to the intensity of felt emotion during both literary reading and music listening. Social cognition comprises just one aspect of these activities, however, and, as hinted at above, the temporal unfolding of “information” over time in the two domains, may have an important influence on the way they are experienced. Accounts of brain function that emphasize prediction and active inference (Friston, 2010) are particularly relevant to dynamically unfolding activities like music listening and reading. Thus, it is interesting to consider how such accounts are informing the investigation of emotional responses to these activities and what the result of such investigations are showing.

## Predicting the Uncertain

In general, both music and language (the building blocks of literature) are comprised of discrete elements that are not combined haphazardly, but according to a set of principles (Patel, 2008). Just as linguistic syntax refers to the rules that guide the way language is constructed, so also has the term *musical syntax* been used to describe the set of principles guiding the combination of musical elements. In the field of cognitive neuroscience, a comparative approach has revealed similar electrophysiological signatures to irregular or unexpected events in the context of music and language (Patel, 2008). Specifically, “mismatch” responses to low probability events (e.g., Koelsch et al., 2001; Omigie et al., 2013) have been associated with longer processing times (Bharucha and Stoeckig, 1986; Omigie et al., 2012) and localized to the left and right inferior frontal gyrus (Maess et al., 2001; Koelsch et al., 2005). At this point it is worth acknowledging that the necessarily short and highly controlled stimuli that have commonly been used to bring about the signature mismatch responses may seem far removed from the rich and complex literary and musical materials experienced in everyday life. However, these mismatch responses have increasingly been interpreted as support for the Bayesian brain hypothesis, which posits that the brain continuously makes active inferences about how events in the environment will unfold (Garrido et al., 2009; Friston, 2010; Gebauer et al., 2012). Critically, growing investigations into the emotional implications of such predictive processes (e.g., Joffily and Coricelli, 2013; Omigie, 2015) raise the possibility that commonly observed electrophysiological responses reflect a broader mechanism underlying our affective responses to a wide range of stimuli.

Recently, attempts to characterize the experience of continuously and actively predicting have moved away from emphasizing correlates of incorrect predictions (as in the electrophysiological responses described above) to emphasizing the state of *uncertainty* experienced as a given sequence unfolds (e.g., Hansen and Pearce, 2014; Lehne and Koelsch, 2015). In a recent comprehensive account, the concept of *Tension* was held to be relevant to music, literature (where tension is referred to as suspense), and a range of other activities, and was operationalized as an emotional experience, accompanying continuous prediction making, that arises from a state of uncertainty and need for resolution (Lehne and Koelsch, 2015). The concept of *Tension* has long been used in music listening (Madsen and Fredrickson, 1993; Bigand and Parncutt, 1999; Lerdahl and Krumhansl, 2007; Farbood, 2012), where its build-up and relief is held to be made possible by listeners’ having internalized the tonal systems and forms of their culture’s music. Importantly, feelings of tension in music have also long been related to changes in physiological responses, for instance in response to increased harmonic complexity (e.g., Krumhansl, 1997; Steinbeis et al., 2006). However, only recently, have the neural correlates of musical tension been directly examined using neuroimaging methods (Lehne et al., 2014; see Koelsch, 2014). Indeed, while Koelsch et al. (2008) had demonstrated that structures like the amygdala and OFC are involved in the processing of syntactically irregular musical events (that brought about the previously mentioned mismatch responses), it was also of interest to see that such structures may be linked to the subjective feelings of musical tension (Lehne et al., 2014). Specifically, it was shown that that continuous subjective ratings of tension as provided by participants, correlated with unfolding activity in left pars orbitalis, an area associated with both predictive and affective processing. Further, a region-of-interest analysis was able to confirm the role of amygdala in mediating feelings of increasing relative to decreasing tension during music listening (Lehne et al., 2014). Interestingly, a number of other studies have also been able to indirectly associate subcortical and limbic structures with uncertainty and anticipation in music (Salimpoor et al., 2011; Trost et al., 2012). For instance, Trost et al. (2012) described neural activity in response to a “tension” emotion (characterized by high arousal, negative valence, and unpredictability) not only in sensory and motor areas (linked to prediction making; Schubotz, 2007) but also in structures like the parahippocampal gyrus and caudate nucleus.

Suspense, the concept equivalent to musical tension in its induction of feelings of uncertainty and anticipation (Lehne and Koelsch, 2015), is held to constitute a critical component of narrative literature (Zillmann, 1980; Brewer and Lichtenstein, 1982; Comisky and Bryant, 1982; Oatley, 1994). Further, like musical tension, it has been shown to modulate physiological responses (Zillmann et al., 1975). Thus, of considerable interest was whether suspense evoked by literary reading would also activate the limbic and deep subcortical structures associated with musical tension (Koelsch et al., 2008; Lehne et al., 2014). In a first ever attempt to isolate the neural correlates of suspense that emerges as participants read a literary text for the first time, the authors presented participants with a narrative broken up into numerous shorter segments while their haemodynamic responses were measured (Lehne et al., 2015). Participants were required to rate each segment, following its presentation, for subjective feelings of suspense. Consequently a parametric regressor that summarized these ratings across participants was used to identify suspense-associated brain regions. The findings were interesting in implicating areas that have been associated with predictive processing in a range of contexts (e.g., inferior frontal gyrus and lateral premotor cortex, see Schubotz, 2007). Further, they were interesting in confirming the role, during the reading of literary texts, of brain areas related to mentalising (e.g., mPFC and TPJ). However, in not implicating a role of subcortical limbic structures in literary tension (as was seen in musical tension), the study from Lehne et al. (2015) suggested differences in the nature of musical and literature-induced uncertainty. Specifically, it suggested differences in the extent of these art forms’ recruitment of evolutionary ancient parts of the emotion-processing network.

It remains possible that any conclusions that may be drawn from the studies reviewed above will be moderated following future research. Indeed an important limitation of the literary tension study from Lehne et al. (2015) was the interrupted way in which the stimuli were presented, namely, in segments rather than all at once as in the musical tension study. It remains possible that these interruptions compromised the affective power of the narrative stimuli and, consequently, the extent to which limbic structures could be recruited. Indeed, as seen in the research reviewed earlier, several studies have been able to show a link between limbic activity and perceived emotional intensity of literary stimuli (Ferstl et al., 2005; Wallentin et al., 2011; Altmann et al., 2012; Nummenmaa et al., 2014). Further, there is compelling evidence of the recruitment of limbic regions during the processing of literary stimuli that have been rendered more complex using artistic devices. Here, it is important to point out that in addition to those states of uncertainty that arise from following a plot in the many literary genres that employ suspense (e.g., crime novels, thrillers), states of uncertainty may also arise from a writer’s use of literary techniques, of which *defamiliarization* is one (van Peer, 1986; Oatley, 1994; Giora et al., 2004). Defamiliarization is defined as the process whereby a writer makes the familiar unfamiliar and has been shown to reduce the overall predictability of a text, while increasing its perceived esthetic value (Miall and Kuiken, 1994; Hanauer, 1998). In a recent imaging study, evidence was sought of a contribution of defamiliarization to the affective and esthetic perception of written words (Bohrn et al., 2012). Interestingly, it was shown that defamiliarized proverbs, such as “Time eats money” (a variant of Time *is* money) increased activity not just in syntax and semantics related brain areas, but also in limbic structures like the amygdala and medial OFC. Such findings suggest that even if uncertainty in the unfolding of a plot may not implicate the limbic network to the same degree as uncertainty in music, literature’s artistic use of language may provide a rich additional source of emotional power.

## Conclusion

In sum, the research literature provides an ever-increasing body of support for the notion of a role of empathy processes during both music listening and literary reading. It also suggests an important role of predictive processes during the consumption of such stimuli, although of interest will be to explore the extent to which uncertainty in the two domains is bound (or not) to activity in the core limbic network. In general, it may be concluded that a comparison of research findings from music and literature focused studies will continue to be enlightening, and that particularly important insights will emerge when studies in the two domains use similar concepts. Critically, it may be expected that while observed overlaps may help to explain the common appeal of music and literature as art forms, differences may help to explain any idiosyncrasies in their respective capacities for affective impact.

### Conflict of Interest Statement

The author declares that the research was conducted in the absence of any commercial or financial relationships that could be construed as a potential conflict of interest.
